# Inherited thrombophilias and stillbirth: a systematic review and meta- analysis

**DOI:** 10.1007/s00404-025-07989-6

**Published:** 2025-03-14

**Authors:** Michail Delis, Elpida Emmanouilidou-Fotoulaki, Christos Chatzakis, Theodoros Theodoridis, Alexandros Sotiriadis

**Affiliations:** 1 1st Department of Obstetrics and Gynecology, Aristotle University of Thessaloniki, Papageorgiou General Hospital, Ring Road, Nea Efkarpia, 54603 Thessaloniki, Greece; 2 1st Department of Pediatrics, Aristotle University of Thessaloniki, Ippokratio General Hospital, 54642 Thessaloniki, Greece; 3 2nd Department of Obstetrics and Gynecology, Aristotle University of Thessaloniki, Ippokratio General Hospital, 54642 Thessaloniki, Greece

**Keywords:** Stillbirth, Intrauterine Death, Thrombophilia, FVL, Prothrombin

## Abstract

**Purpose:**

The association between inherited thrombophilias and stillbirth has been long investigated but the estimated risk remains unknown. The aim of our study is to summarize available data on the effect of Factor V Leiden, Prothrombin G20210A and MTHFR mutation, Protein S, Protein C and Anithrombin deficiency on the prevalence of stillbirth.

**Methods:**

We conducted a systematic review and meta- analysis of all relevant available PubMed, Embase and Cochrane studies until February 2024. A sensitivity analysis of only prospective and retrospective studies was performed.

**Results:**

Based on 31 included studies, Factor V Leiden and Prothrombin G202110A mutations, significantly rise the prevalence of stillbirth with a pooled OR 2.35 (95% CI 1.74–3.17) and 2.62 (95% CI 1.79–3.84), respectively. This positive correlation did not change in the sensitivity analysis. Positive correlation was also found between Antithrombin deficiency and stillbirth with a pooled OR 3.97 (95% CI 1.50–10.48). No statistically significant relationship was found between stillbirth and MTHFR mutation or Protein C and Protein S deficiency according to the random effects model.

**Conclusion:**

Our findings suggest that in the presence of certain inherited thrombophilias, the occurrence of intrauterine fetal death is significantly more prevalent.

**Supplementary Information:**

The online version contains supplementary material available at 10.1007/s00404-025-07989-6.

## Introduction

Intrauterine death is probably the most catastrophic event that can occur in a pregnancy. Identification and treatment of risk factors for stillbirth as well as management of high risk pregnancies is a major part of obstetric care. Even though there are several conditions in which fetal death is more prevalent, a great number of stillbirths occur in uncomplicated pregnancies, representing the subgroup of unexplained stillbirth. Data from previous studies suggest that in the presence of some thrombophilias, there is a serious risk for intrauterine fetal death [1–3], which is in many cases classified as unexplained, as those fetuses usually have normal growth and no pathology complicating the pregnancy. Nonetheless, there is no consensus in the guidelines of the biggest colleges whether the existence of inherited thrombophilias is a risk factor that should be assessed in pregnancies, even after an event of unexplained fetal death [4, 5]. Therefore, the question remains. The role of inherited thrombophilias in the occurrence of intrauterine death has been investigated in many studies since the 1990s’ and new data from recent well designed cohorts have come up after the last meta-analyses more than ten years ago [2, 6]. The most common inherited thrombophilic disorders in everyday practice are Factor V Leiden mutation (FVL), Prothrombin G20210A mutation (PGM), MTHFR mutation homozygosity, and protein C (PCD), Protein S (PSD) and Antithrombin (ATD) deficiency. The aim of our study is to analyze all available data and provide up to date data concerning this association.

## Materials and methods

### Study registration and search methodology

**Fig. 1:**
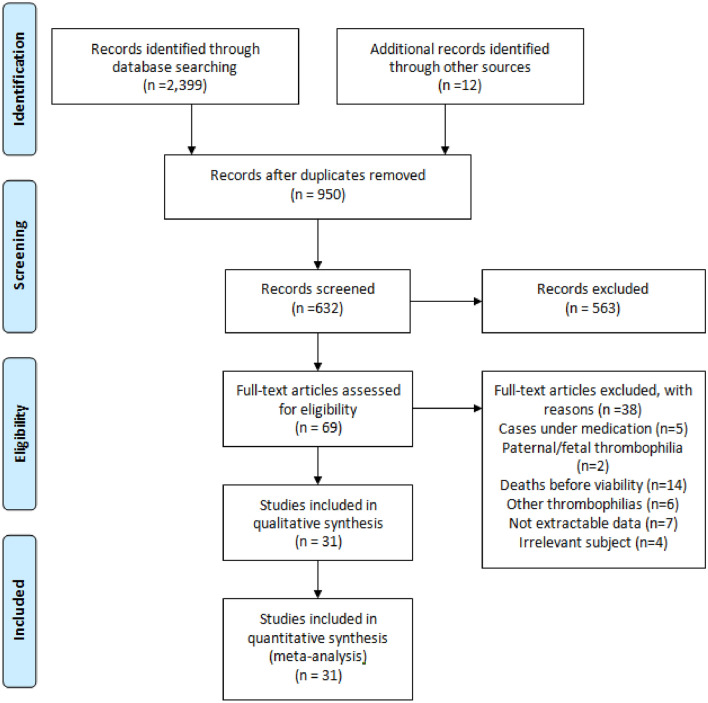
Flowchart

The meta-analysis was conducted according to the Preferred Reporting Items for Systematic Review and Meta-analyses (PRISMA) guidelines and the Cochrane Handbook for Systematic Reviews of Interventions [7, 8]. A preconceived protocol has been registered in PROSPERO (ID number: CRD42024507063). An extensive literature search was conducted by two individual reviewers in EMBASE, MEDLINE, and COCHRANE databases until February 2024. The MESH terms used in our search were: Thrombophilia, FVL, Prothrombin AND stillbirth, intrauterine death and fetal death in all their possible compilations. There was no limitation for publication year. We also screened all the references of selected articles and 12 additional studies were included as shown in Fig. 1. Duplicates were deleted resulting in the final sample included in our systematic review and meta-analysis.

### Inclusion and exclusion criteria

In our study, we included articles in English language, studies referring to humans, that associated the outcome with maternal thrombophilia and not paternal or fetal thrombophilias. We excluded studies in which the participants were under aspirin or low molecular weight heparin medication and those that did not present their data in a form that they could be extracted for analysis. We also only aimed in articles which defined that fetal deaths happened after the limit of viability.

### Study procedure and data extraction

After the exclusion of duplicated reports, case reports, letters, animal studies, studies in other language and reviews (using the Rayyan AI tool [9]), two independent reviewers applied the inclusion and exclusion criteria on the remaining articles and full-text reading of retrieved records was performed independently. Upon disagreement there was consensus after discussion. Data concerning the presence of thrombophilias and fetal deaths in carriers and non carriers, as well as the limit of viability and the exclusion criteria for the population of each study were extracted independently using structured forms. Most of the studies separated stillbirths in categories according to whether the pregnancy was normal in terms of obstetric conditions, fetal anomaly, delivery conditions or maternal history of health problems. We aimed only to include unexplained stillbirths in the study. When a study separated stillbirth in explained (attributed to other known conditions) and unexplained, only unexplained were included in the study’s population. In cases where information about the cause was not specified, the total sample was included. Quality assessment was done independently using the QUIPS tool. All disagreements were discussed between the reviewers in the end of the data extraction procedure.

### Quality assessment

Risk of bias (RoB) was assessed by the two reviewers independently. The assessment of risk of bias (RoB) was conducted using the Quality in Prognosis Studies (QUIPS) tool [10], recommended by the Cochrane Prognosis Methods Group. It uses six important domains that should be critically appraised when evaluating validity and bias: 1) The risk of selection bias, 2) the risk of attrition bias, 3) the risk of measurement bias related to how the prognostic factor is measured, 4) risk of bias related to the measurement of the outcome, 5) bias due to confounding, and 6) bias related to statistical analysis and presentation of the results. The results for each study are shown in Table 1. A copy of QUIPS as well as the results graph is included in the supplementary material (Figs. 21–24).

**Table 1:** Studies included in the meta-analysis

Study	Design	Thrombophilia	Participants	SB Definition	Excluded	Qualiy assessment
Abu-Asab 2011 [56]	Case–control	FVL, PGM, MTHFR C667T	65 Palestinian women with SB 402 controls non pregnant, at least one healthy infant	Any “child” expelled from its mother at 24th week of pregnancy or more without showing any sign of life	PI, ID, TD, UM, CH	1 low, 2 low, 3 low, 4 low, 5 moderate, 6 low
Agorastos 2002 [57]	Case–control	FVL, PGM	8 women with SB. 100 matched women with at least one uneventful pregnancy and no history of thrombosis	Fetal death after 24 weeks of gestation	CA, CI, AI, GI	1 low, 2 low, 3 low, 4 low, 5 low, 6 low
Alfirevic 2001 [66]	Case–control	FVL, PGM, MTHFR C667T, PCD, PSD, ATD, homocystein, presence of lupus anticoagulant and anticardiolipin antibodies	18 women who had unexplained stillbirth and 44 healthy postpartum women with uncomplicated pregnancies, with no abnormal medical or obstetric history, matched	Unexplained stillbirth after 23 completed weeks of gestation	CA, TD	1 low, 2 low, 3 low, 4 low, 5 moderate, 6 low
Alonso 2002 [67]	Case–control	FVL, PGM, MTHFR C667T, PCD, PSD, ATD, homocystein, presence of lupus anticoagulant and anticardiolipin antibodies	8 women who had ≥ 1 unexplained SB and 75 healthy control subjects with at least one successful pregnancy and no history of thrombosis or fetal loss	Intrauterine fetal death defined according to the criteria of the World Health Organization at ≥ 23 weeks	CD, CA, CI, ED, UM, TD, ND	1 low, 2 low, 3 low, 4 low, 5 low, 6 low
Bare 2000 [76]	Retrospective cohort	FV Leiden	128 female Leiden mutation carriers (including 4 homozygotes) and 461 control females	Intrauterine death (week not stated) distinguished from abortions	NONE	1 low, 2 low, 3 low, 4 low, 5 moderate, 6 low
Bjork 2019 [6]	Case–control	FV Leiden	Cases 963 singleton SB tested for FVL. Controls unselected group of 1762 women with no history of thrombosis	Stillbirth at ≥ 22 + 0 weeks of gestation	TD, MP	1 low, 2 low, 3 low, 4 low, 5 moderate, 6 low
Clark 2008 [58]	Prospective cohort	FV Leiden	3944 pregnancies tested for FVL 22 SB	Stillbirth at ≥ 24 weeks of gestation	NONE	1 low, 2 low, 3 low, 4 low, 5 moderate, 6 low
Gonen 2005 [70]	Case–control	FVL, PGM, MTHFR C667T, PCD, PSD, ATD,FVII, FXI, lupus anticoagulant, b2-glycoprotein, anticardiolipin antibodies	37 women with a history of unexplained SB, with no risk factors for SB. Controls 46 women with history of at least one live birth with no history of SB or VTE	Stillbirth at 27–42 weeks of gestation	MP, CA, CI, HF, HT, GI, TD, ID	1 low, 2 moderate, 3 low, 4 low, 5 low, 6 low
Gris 1999 [62]	Case–control	APCR, PGM, MTHFR C667T, PCD, PSD, ATD, presence of lupus anticoagulant and anticardiolipin antibodies	232 women with one or more unexplained singleton stillbirth. Controls 464 matched women with normal pregnancies	Stillbirth at ≥ 22 weeks of gestation	TD, CA, HT, CI, HF, I.T.P., F.A.T	1 low, 2 low, 3 low, 4 low, 5 low, 6 low
Hefler 2004 [48]	Case–control	FVL, FV H1299R, PGM, FXIII V34L, MTHFR C667T, MTHFR A1298C, beta-fibrinogen -455 G to A, GPIIIa L33P), PAI-1 4G/5G, HFEC282Y, apolipoprotein B R3500Q and apolipoprotein E2/E3/E4	94 women with unexplained singleton stillbirth. Controls 94 women with at least one uncomplicated full-term pregnancy delivering normally	Stillbirth at ≥ 20 weeks of gestation	TD, PPROM, PL, CD, CI, MP, HF	1 low, 2 low, 3 low, 4 low, 5 low, 6 low
Helgadottir 2011 [68]	Case–control	FVL, PGM, MTHFR C667T, PCD, PSD, ATD	105 unselected cases with history of IUFD, 262 unselected controls with live births	Intrauterine fetal death at ≥ 23 completed gestational weeks or birth weight greater than 500 g	2 women because of warfarin use	1 low, 2 low, 3 low, 4 low, 5 moderate, 6 low
Hiltunen 2010 [64]	Case–control	FV Leiden, PGM MTHFR C667T	44 cases of unexplained singleton stillbirth and 766 controls	Stillbirth at ≥ 22 weeks of gestation	CD, CA CC, CI, AI	1 low, 2 moderate, 3 low, 4 low, 5 low, 6 low
Kaur 2013 [59]	Case–control	FVL, MTHFR C667T	66 women with SB. Matched controls	Intrauterine death (week not stated) distinguished from abortions (≤ 24 weeks)	None	1 low, 2 low, 3 low, 4 low, 5 moderate, 6 low
Kjellberg 2010 [63]	Prospective case–control	FV Leiden	491 singleton FVL carriers, 0 unexplained SB and 1055 controls 1 unexplained IUFD	Stillbirth at ≥ 22 weeks of gestation	Aspirin/heparin use, TD, CD, MP, PPROM	1 low, 2 low, 3 low, 4 low, 5 low, 6 low
Kocher 2007 [75]	Prospective case–control	FVL, PGM	4872 women tested, 32 SB in white women	Dead fetuses or neonates ≥ 500 g	Trauma, age not 17–45, IC, vWD, TD, thalassemia, black	1 low, 2 low, 3 low, 4 low, 5 moderate, 6 low
Kovac 2010 [49]	Case–control	FVL, PGM, MTHFR C667T, PCD, PSD, ATD, Antiphospholipid antibodies	55 unexplained SB. Control group 128 healthy women, with no previous history of miscarriages or thrombotic events	Stillbirth at ≥ 20 weeks of gestation	CD, UM, HD, GI, TD, CI, ID, HT	1 low, 2 low, 3 low, 4 low, 5 moderate, 6 low
Kupferminc 1999 [69]	Case–control	FVL, PGM, MTHFR C667T, PCD, PSD, ATD, anticardiolipin antibodies	12 unexplained SB. Controls 110 women with one or more normal pregnancies	Stillbirth at ≥ 23 weeks of gestation	CD, CA, CI, AI, GI	1 low, 2 low, 3 low, 4 low, 5 low, 6 low
Lindqvist 2006 [71]	Prospective cohort	FVL, APCR	2480 unselected gravidae checked for APCR and FVL at 12 weeks gestation	Stillbirth at ≥ 27 weeks of gestation	NONE	1 low, 2 low, 3 low, 4 low, 5 moderate, 6 low
Many 2002 [72]	Case–control	FVL, PGM, MTHFR C667T, PCD, PSD, ATD, anticardiolipin antibodies	40 women with unexplained singleton fetal death. Controls 80 matched healthy women with at least one normal pregnancy	Stillbirth at ≥ 27 weeks of gestation	MP, CA, CI, HF, GI, HT, TD	1 low, 2 low, 3 low, 4 low, 5 low, 6 low
Martinelli 2000 [50]	Case–control	FVL,PGM, MTHFR C667T	67 women with a first episode of unexplained late fetal loss. Controls 232 women who had had one or more normal pregnancies and no late fetal losses	Stillbirth at ≥ 20 weeks of gestation	Previous late loss, UM, Abnormal placental insertion, drug abuse, CA, CD, HF, AI, Dead Twin	1 low, 2 low, 3 low, 4 low, 5 low, 6 low
Meinardi 1999 [51]	Retrospectivecohort	FV Leiden	228 carriers of the factor V Leiden mutation. Controls 121 non carrier relatives. All participants had been pregnant at least once	Stillbirth at ≥ 20 weeks of gestation	NONE	1 low, 2 low, 3 low, 4 low, 5 moderate, 6 low
Monari 2012 [65]	Consecutive prospective case–control	FVL, PGM, anticardiolipin antibodies and lupus anticoagulants	171 cases of antenatal SB (79 unexplained). 326 controls with uneventful pregnancy matched for age and ethnicity	Stillbirth at ≥ 22 weeks of gestation or 500 g if gestational ultrasound age was not avail able	Totally unexplained when not CA, CD, CI, HF, TTTS, SGA, PE, AP, HT	1 low, 2 low, 3 low, 4 low, 5 low, 6 low
Nurk 2006 [77]	Population-based study	FVL, MTHFR C667T	5874 women with 14 474 pregnancies checked	SB weeks not defined	NONE	1 low, 2 low, 3 low, 4 low, 5 moderate, 6 low
Preston 1996 [73]	Retrospective cohort	FVL, PCD, PSD, ATD	571 women with 1524 pregnancies positive for thrombophilia by EPCOT study. Control women 395 with 1019 pregnancies, partners of male members of the EPCOT cohort or acquaintances of cases	Stillbirth at ≥ 28 weeks of gestation	NONE	1 low, 2 low, 3 low, 4 low, 5 moderate, 6 low
Rothbart 1999 [60]	Case–control	FV Leiden	14 non-pregnant women with a history of unexplained intrauterine fetal death and 14 healthy controls matched	Stillbirth at ≥ 24 weeks of gestation	Maternal disease, AP, CC, IUGR, CI, CA, TD	1 low, 2 low, 3 low, 4 low, 5 low, 6 low
Said 2010 [52]	Prospective cohort	FV Leiden, PGM, MTHFR C677T, MTHFR A1298C, and thrombomodulin polymorphism	1707 Healthy nulliparous women recruited prospectively before 22 weeks of gestation (SB 20 weeks)	Birth of a fetus weighing at least 400 g or at least 20 weeks of gestation that shows no signs of life after birth	MP, CA, HT, RPL, SLE, drug abuse, renal disease, preexisting diabetes	1 low, 2 low, 3 low, 4 low, 5 moderate, 6 low
Sottilotta 2006 [55]	Case–control	FVL, PGM	47 women with unexplained SB studied for hereditary thrombophilia. Control group 217 healthy women from the general population with at least one normal pregnancy	Stillbirth at ≥ 20 weeks of gestation	UM, Placental insertion, CD, Drug abuse, TD	1 low, 2 low, 3 low, 4 low, 5 low, 6 low
Simchen 2010 [54]	Case–control	FVL, PGM, MTHFR C667T, PCD, PSD, ATD, circulating anticoagulant, anticardiolipin antibodies and beta-2 glycoprotein 1 antibodies	Cases 67 women with SB (33 placental) Controls were 637 low risk nulliparous women from another study	Stillbirth at ≥ 20 weeks of gestation	GI, HT, CI, AI, CC, CA	1 low, 2 low, 3 low, 4 low, 5 low, 6 low
Silver 2016 [53]	Secondary analysis case–control	FVL, PGM, MTHFR C667T, MTHFR A1298C, PAI 4G/5G	Cases 359 with singleton SB, excluded intrapartum deaths and anomalies. Controls 1303 women. All from SCRN study	Apgar scores of 0 at 1 and 5 min and no signs of life by direct observation at ≥ 20 weeks of gestation and 18 weeks when bad dating	MP, CA, CD, 13 years old	1 low, 2 low, 3 low, 4 low, 5 low, 6 low
Volkze 2003 [74]	Cross sectional	FVL	1768 women with at least one pregnancy. 1657 FVL negative, 111 positive. 73 with at least one SB	Stillbirth at ≥ 28 weeks of gestation	NONE	1 low, 2 low, 3 low, 4 low, 5 moderate, 6 low
Weiner 2004 [61]	Case–control	FVL, PGM, MTHFR C667T, PCD, PSD, ATD, presence of lupus anticoagulant and anticardiolipin antibodies	Cases 53 women with unexplained SB. Controls 59 women with unremarkable obstetric history who delivered at the same period	Stillbirth at ≥ 24 weeks of gestation	MP, CA, CD, HF, AI, CC, post term, uncontrolled diabetes,	1 low, 2 low, 3 low, 4 low, 5 low, 6 low

*FVL* factor V leiden, *PGM* prothrombin gene mutation, *MTHFR* Methylenetetrahydrofolatereductase, *PSD* protein S deficiency, *PCD* protein C deficiency, *ATD* antithrombin deficiency, *SB* stillbirth, *IUD* intrauterine death, *APCR* activated protein C resistance, *PAI* plasminogen activator inhibitor, *PI* prior infertility, *ID* immunological disease, *TD* thrombophilic disorder, *UM* uterine malformation, *CD* chromosome disorder, *CA* congenital anomaly, *CI* congenital infection, *GI* glucose intolerance, *AI* alloimmunization, *ED* endocrine disorder, *ND* neoplastic disease, *MP* multiple pregnancy, *HF* hydrops fetalis, *HT* hypertension. *F.A.T.* foetomaternal alloimmune thrombocytopenia, *I.T.P.* immune thrombocytopenic purpura, *PL* premature labor, *CC* umbilical cord complications, *IC* incompetent cervix, *HD* hormone disorder, *TD* thyroid disorder, *AP* abruptio placentae, *PE* preeclampsia, *RPL* recurrent pregnancy loss

Quality assessment was done using the QUIPS tool

### Statistical analysis

R version 4.1.2 for Windows (R Core Team, Vienna, Austria) was used for the statistical analysis of the meta-analysis. At least three studies were required to combine data on outcomes and that was permitted for all thrombophilic defects. Heterogeneity between the studies was assessed using the *I*^2^ test or Q test. *I*^2^ value < 40% was considered low, 30–60% as moderate, 50–90% as substantial and 75–100% as considerable. Due to the design of the studies (non-randomized observational studies) and the different clinical criteria of each study, the expected heterogeneity between studies is large making it more appropriate to use random effects models. Random effects model assumes that the true effect size is not constant but follows a normal distribution and each individual study is a random sample of the possible observed sizes. It, therefore, computes two values ​​of variability, the random sampling error of each study and the expected variability between studies. It thus provides more conservative estimates with wider 95% CIs. The number of intrauterine deaths, live births and women with inherited thrombophilias were collected from each study. Meta-analysis was performed using logarithmic Odds Ratio (OR) for each study. Pooled effect measure was calculated as Odds Ratio (OR) using the inverse-variance method and the restricted maximum-likelihood estimator for all thrombophilic defects. A P-value threshold for statistical significance was set at 0.05 and data were analyzed using 95% confidence interval (95% CI). Finally, a leave-one-out influence analysis was conducted, omitting each study consecutively to explore its effect on the overall outcome. Sensitivity analysis was performed based on study design. Pooled effect size of prospective and retrospective studies was presented separately in a sensitivity analysis to check if the methodology of the study has a role in the outcome.

## Results

Our initial search ended in assembling 2399 from database searching and 12 from the articles’ references (Fig. 1). An open AI tool «RAYYAN»(9) was used to delete duplicates resulting in 950 articles and after exclusion of reviews, animal studies, case reports, we came up with 632 original articles for title and summary screening. 69 of those were found compatible for full text reading. After removing 5 studies in which pregnant women were under medication [11–15], 2 studies referring to fetal or paternal thrombophilias [16, 17], 14 studies with fetal diminish before viability age [18–31], 6 studies who assessed different thrombophilias than those of our interest [32–37], 7 studies with no extractable data [38–43] and 4 studies with irrelevant subject to our review [44–47], we ended up with our 31 studies to be concluded for meta-analysis. The whole process is shown in Fig. 1 and the basic features of the included studies in Table 1. Stillbirth was defined with different criteria between the studies. The most common was fetal death after 20 weeks [48–55] of gestation in most studies*,* following fetal death after 24 weeks [56–61], 22 weeks [6, 62–65], 23 weeks [66–69], 27 weeks [70–72], 28 weeks [73, 74]. Some studies examined birth weight as a criterion [52, 65, 68, 75] while in two studies neither gestational age nor weight was stated [76, 77].

### FVL and stillbirth

**Fig. 2:**
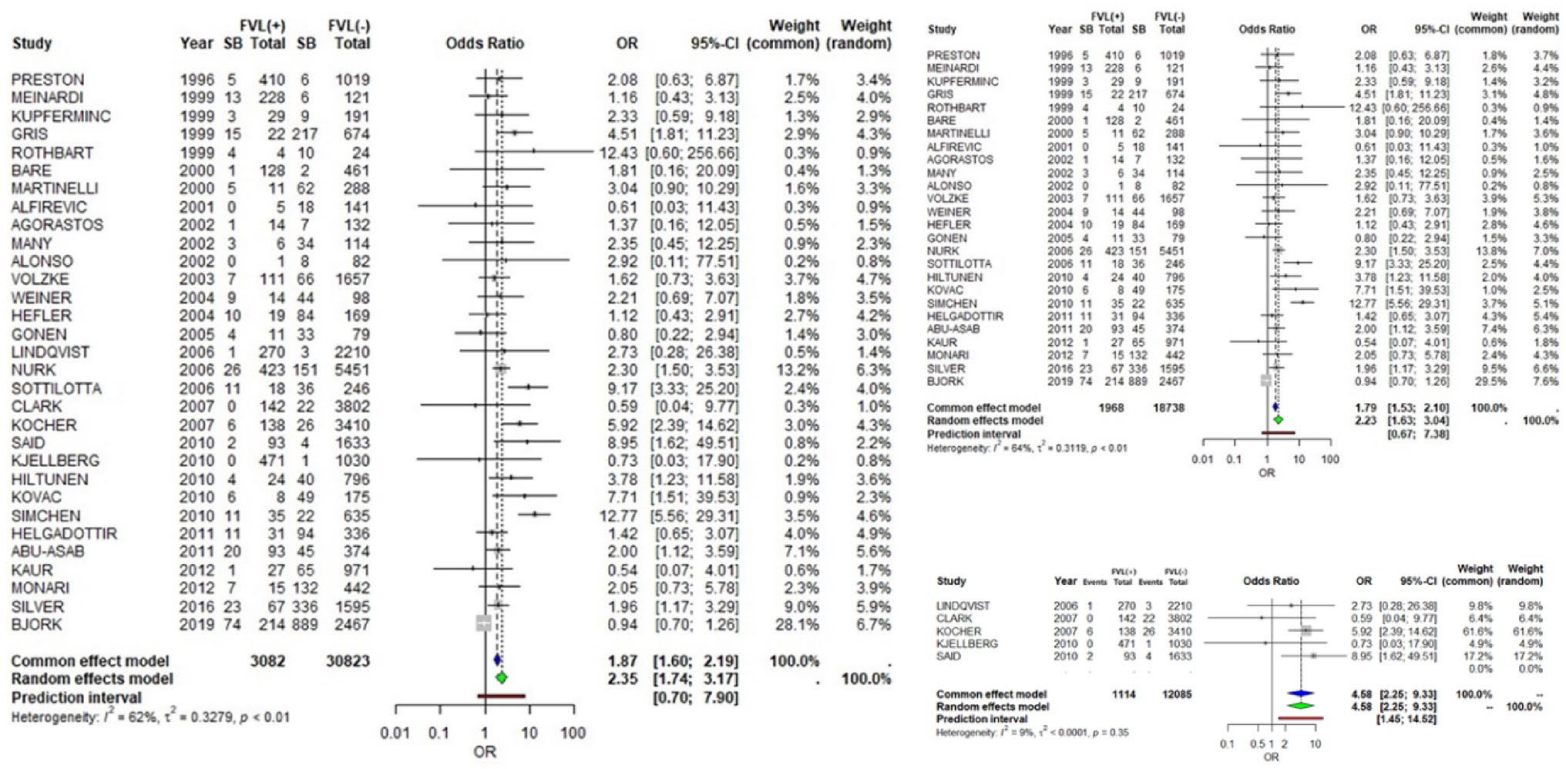
FVL and stillbirth

Data collected from 31 studies show that in the presence of Leiden mutation (FVL), stillbirth is statistically significantly more prevalent with a pooled OR 2.35 (95% CI 1.74–3.17, *Ι*^2^ = 62%, *p*-value < 0.01) using the random effects model. Interestingly, the result remains constant in the sensitivity analysis; this association does not seem to change according to the study design, with a pooled OR 4.58 (95% CI 2.25–9.33, *I*^2^ = 9%, *p*-value = 0.35) in prospective studies, and pooled OR 2.23 (95% CI 1.63–3.04, *Ι*^2^ = 64%, *p*-value < 0.01) in retrospective studies (Fig. 2).

### PGM and stillbirth

**Fig. 3:**
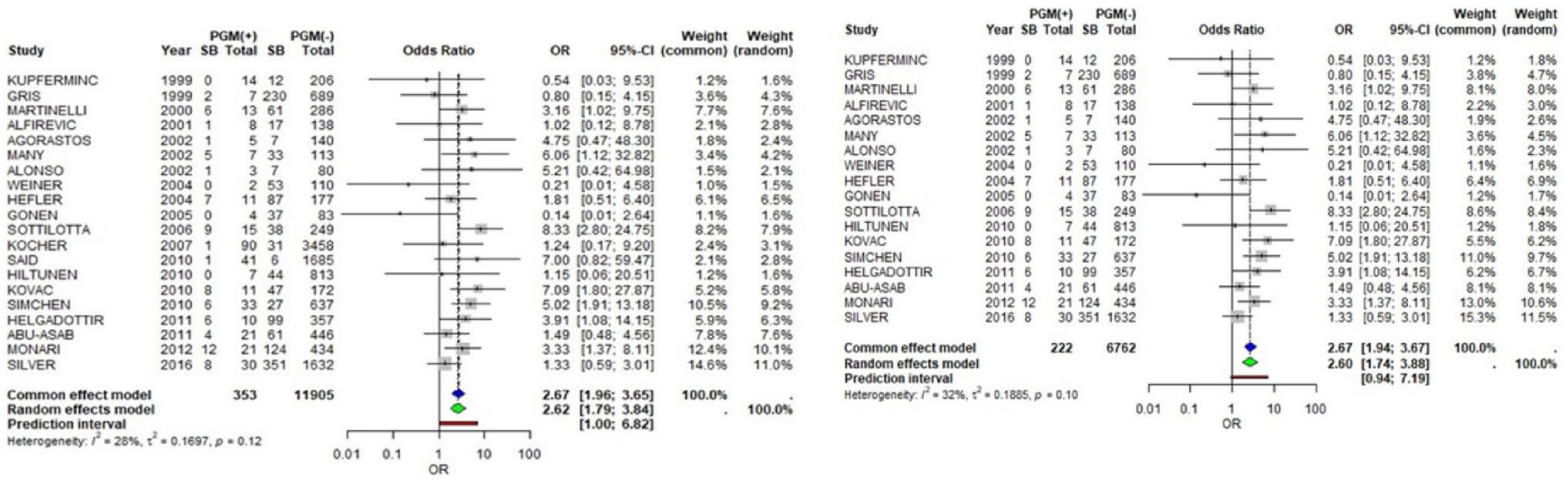
PGM and stillbirth

Regarding Prothrombin G20210A mutation, we collected data from 20 studies, 2 prospective and 18 retrospective. According to the random effects model, in the presence of G20210A mutation, the occurrence of intrauterine fetal death is statistically significantly more prevalent with a pooled OR 2.62 (95% CI 1.79–3.84, *Ι*^2^ = 28%, *p*-value = 0.12). This association does not seem to change if we leave the prospective studies out of the results in our sensitivity analysis; pooled OR 2.60 (95% CI 1.74–3.88, *Ι*^2^ = 32%, *p*-value = 0.10) (Fig. 3).

### MTHFR and stillbirth

**Fig. 4:**
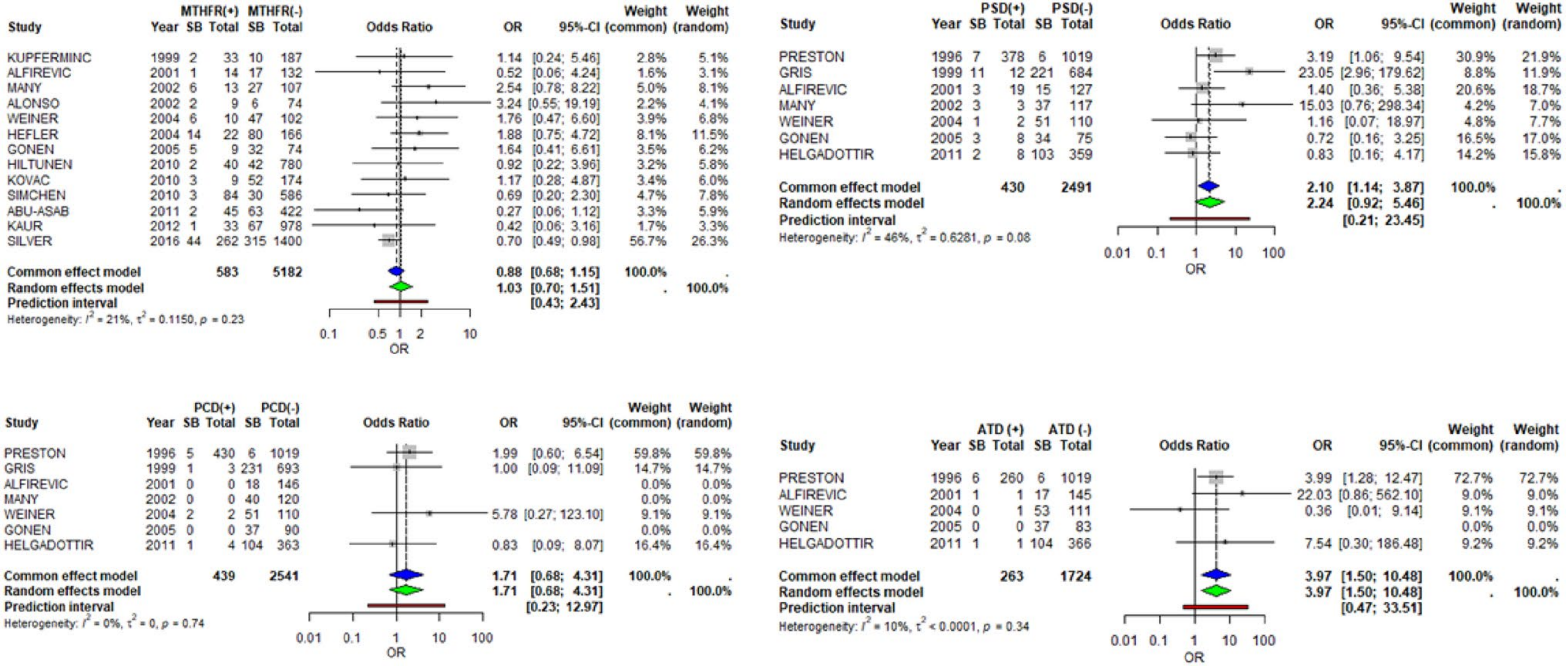
MTHFR, PCD, PSD, ATD and stillbirth

Data from 13 studies referred to MTHFR mutation and stillbirth. According to the random effects model, the presence of MTHFR mutation does not change the risk of fetal death (pooled OR 1.03, 95% CI 0.70–1.51, *Ι*^2^ = 21%, *p*-value = 0.23) (Fig. 4).

### Protein C deficiency and stillbirth

We collected data from four retrospective studies. According to our results, deficiency in protein C does not increase the risk of stillbirth significantly (pooled OR 1.71, 95% CI 0.68–4.31 *Ι*^2^ = 0%, *p*-value = 0.74) (Fig. 4).

### Protein S deficiency and stillbirth

Data from 7 retrospective studies show that in the presence of protein S deficiency, there is a tendency for increase in fetal deaths. Using the random effects model this relationship seems marginally not significant with a pooled OR 2.24 (95% CI 0.92–5.46 *Ι*^2^ = 46%, *p*-value = 0.08) (Fig. 4).

### Antithrombin deficiency and stillbirth

Data from 4 retrospective studies show a statistically significant increase in stillbirths when Antithrombin deficiency is present (pooled OR 3.97, 95% CI 1.5010.48 *Ι*^2^ = 10%, *p*-value = 0.34 > 0.05) (Fig. 4).

### Risk of bias assessment

The assessment of systematic bias was done using the QUIPS tool answering the six questions about: (1) The risk of selection bias. (2) The risk of attrition bias. (3) The risk of measurement bias related to how the prognostic factor is measured. (4) Risk of bias related to the measurement of the outcome. (5) Bias due to confounding. (6) Bias related to statistical analysis and presentation of the results. The results are shown on figure A17 (supplementary material).

### Leave-one-out analysis

A leave-one-out sensitivity analysis was performed for each outcome excluding each study consecutively to investigate the existence of any significant effect on the pooled estimate. Only after the exclusion of the (Preston et al. 1996 [73]) study from the pooled effect of Antithrombin III, a non-statistically significant effect emerged on the occurrence of intrauterine death (OR 3.92, 95% CI 0.35–43.32). No other significant changes were noted. The results are shown in detail in the supplementary material (figures A1-A10).

### Publication bias assessment

Funnel plot asymmetry and Eggers test were used to assess publication bias although the statistical power of these tests may not be high enough to detect real asymmetry in small samples. With a visual interpretation of the funnel plots (supplementary material figures A11-A16) and Egger’s regression test, we concluded that no increased risk for publication bias is raised regarding the studies included in this meta-analysis.

## Discussion

Our results suggest that in the presence of factor V Leiden and prothrombin G20210A mutations, the risk of fetal death rises significantly. This correlation remained in our sensitivity analysis of only prospective and only retrospective studies. A significant rise in the risk of fetal death was also found in pregnant women with antithrombin deficiency. In patients with protein S deficiency our results show a marginally not statistically significant increase of the risk, while the MTHFR mutation and protein C deficiency does not seem to raise the risk for stillbirth.

Data from previous meta-analyses also show results in the same direction. Rey et al. [1] found that the risk of fetal loss after 19 weeks of gestation was greater in women with the FVL mutation OR 3.26 (95% CI 1.82–5.83) as do the results of Robertson et al. [3] and Rodger et al. [2] OR 2.6 (95% CI 1.1–3.86) and OR 1.52 (95% CI 1.06–2.19), respectively. The risk of stillbirth in prothrombin gene mutation in Rey’s study was OR 2.3 (95% CI 1.09–4.87) and in Robertson’s studies OR 2.66 (95% CI 1.28–5.53) results similar to ours. Our findings referring to the MTHFR mutation bearers also seem to agree. Results referring to the other inherited thrombophilias seem to differ from the previous data [3], but the small number of studies measuring those mutations in addition to the rare prevalence of these defects is probably the reason for this differentiation. Nonetheless, we believe that including more studies and thus significantly greater number of participants in our analysis it is not unexpected for our results to be different.

One factor that should be highlighted is the great heterogeneity between the studies that cannot be fully interpreted. As shown in Table 1, the methods and the population of the included studies vary significantly. Thus, the gestational age of viability which defines fetal death is not the same for all the studies, as in the 23 year difference from the oldest [73] to the most recent [6] cohort, the viability limit had changed. It is also noteworthy that not all the studies tested all participants for all the mutations. Therefore, it is reasonable that some subjects in the control groups were carriers of mutations that they were not screened for. Homocystein levels were not defined in most studies in patients with MTHFR mutation and the borderlines values for defining natural anticoagulant deficiency thrombophilias were also not specifically defined in most of the studies. Similarly, as not all the included studies have the same exclusion criteria, the definition of a normal pregnancy differs among them, resembling another source of heterogeneity. As we analyze in Table 1, some studies exclude conditions such as twin pregnancies, maternal medical conditions like diabetes or thyroid disease, history of venous thrombosis, drug abuse or fetal anomaly, etc. from the study populations, while others do not. The definition of normal pregnancy to enter the control group also changes, as for some authors it is necessary to give birth to a normal weight full term infant without any obstetric complications during the pregnancy, while for others, the labor of a living infant is enough for the pregnancy to be registered as normal control.

There are some theories that try to justify the association between stillbirth and maternal thrombophilia, most of which suggest impaired microvascular circulation due to thrombophilia and thus abnormal placentation. Data from pathology studies comparing placentas of complicated pregnancies from mothers with and without thrombophilia failed to prove that thrombotic placental lesions are more prevalent in women with thrombophilias rather than in those without [78–81]. Data from previous meta-analysis also show controversial results regarding the risk for placental mediated pregnancy disorders such as preeclampsia, placenta abruption and fetal growth restriction in women with thrombophilias [2, 3]. Although Robertson’s meta-analysis suggests that women with FVL and PGM are in greater risk for preeclampsia and placenta abruption, this is not confirmed in Rodger’s meta-analysis. Both reviews however agree that FVL is not associated with a higher risk for FGR which also seems discrepant to the pathophysiologic hypotheses.

Our study as did the previous ones, suggest that in the presence of some inherited thrombophilias, the occurrence of fetal death raises significantly. Homozygous carriers are probably in greater risk for pregnancy complications as it is observed in some studies [53, 54, 61]. However, data referring to homozygotes were scarce, not allowing the reviewers to synthesize them into the meta-analysis. Taking under consideration that in some geographic regiments, the combined prevalence of these mutations can be as high as 10% [82–86], we believe that health care providers should be aware of this correlation when confronting a pregnancy with thrombophilia in their everyday practice and maybe offer those women closer obstetric surveillance. Referring to the MTHFR mutations, data also show great differences between different populations. In a 2015 paper [87], 20–40% of Hispanic and white USA residents are C677T heterozygous carriers and 8–20% homozygous. The mutation is less often found in black individuals (1–2%). 7–12% of North American, European and Australian people are carriers of the A1298C MTHFR mutation, while the mutation is rarer in Hispanics (4–5%) and Chinese and Asian populations (1–4%). These numbers seem in compliance with the prevalence of the mutations in the populations of our included studies with the highest C677T homozygous prevalence among controls being in the Silver 2016 (16.6%) USA and the lowest in Kaur 2012 (3.5%) India.

It is interesting that all major guidelines agree in not scanning for maternal heritable thrombophilia after an event of stillbirth [88], even though in a recent Delphi consensus [89] most of the participating healthcare professionals with an active interest in stillbirth prevention or prediction classified these thrombophilias as a significant risk factor for fetal death. Larger and better designed studies would probably help to reduce the problem of heterogeneity and lead to safer conclusions about the existence and causes of this condition, and probably suggest different management options that could reduce the risk of stillbirth.

## Supplementary Information

Below is the link to the electronic supplementary material.

Supplementary file1 (PNG 46 KB)

Supplementary file2 (PNG 54 KB)

Supplementary file3 (PNG 125 KB)

Supplementary file4 (PNG 67 KB)

Supplementary file5 (PNG 59 KB)

Supplementary file6 (PNG 43 KB)

Supplementary file7 (PNG 43 KB)

Supplementary file8 (PNG 259 KB)

Supplementary file9 (PNG 243 KB)

Supplementary file10 (PNG 250 KB)

Supplementary file11 (PNG 222 KB)

Supplementary file12 (PNG 209 KB)

Supplementary file13 (PNG 45 KB)

Supplementary file14 (PNG 208 KB)

Supplementary file15 (PNG 91 KB)

Supplementary file16 (PNG 98 KB)

Supplementary file17 (PNG 67 KB)

Supplementary file18 (PNG 49 KB)

Supplementary file19 (PNG 67 KB)

Supplementary file20 (PNG 62 KB)

Supplementary file21 (PNG 47 KB)

## Data Availability

No datasets were generated or analysed during the current study.
