# Supplementary figures and images for: Inherited thrombophilias and stillbirth: a systematic review and meta- analysis
Source: Arch Gynecol Obstet. 2025 Mar 14;312(1):37–50. doi: 10.1007/s00404-025-07989-6 (PMC12177024; doi:10.1007/s00404-025-07989-6)

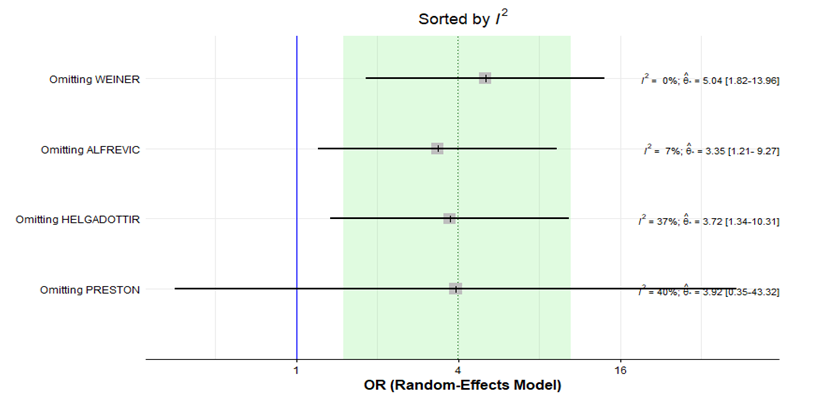

Supplement: Supplementary file 1 — Supplementary file1 (PNG 46 KB) [file 404_2025_7989_MOESM1_ESM.png]

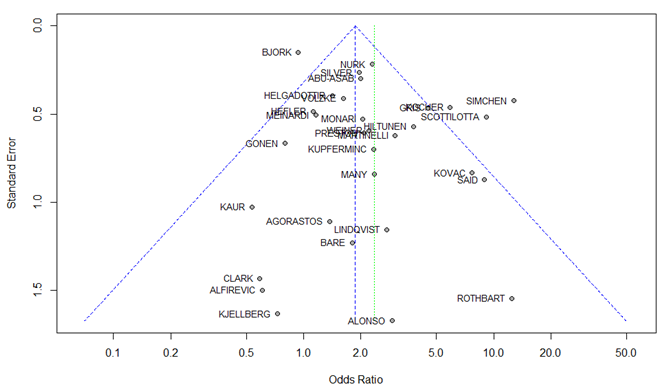

Supplement: Supplementary file 2 — Supplementary file2 (PNG 54 KB) [file 404_2025_7989_MOESM2_ESM.png]

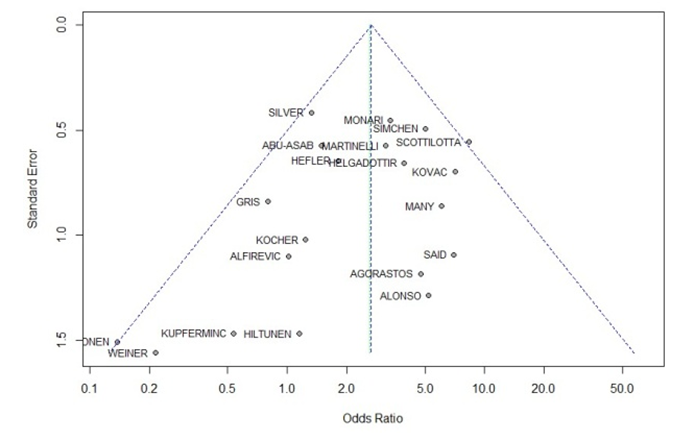

Supplement: Supplementary file 3 — Supplementary file3 (PNG 125 KB) [file 404_2025_7989_MOESM3_ESM.png]

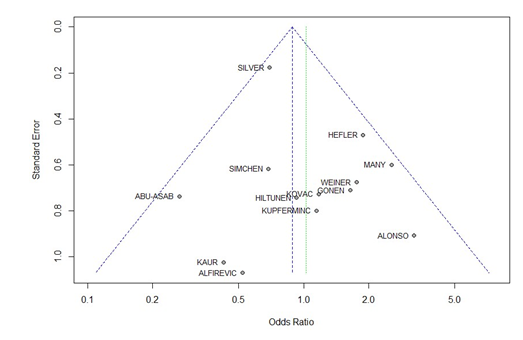

Supplement: Supplementary file 4 — Supplementary file4 (PNG 67 KB) [file 404_2025_7989_MOESM4_ESM.png]

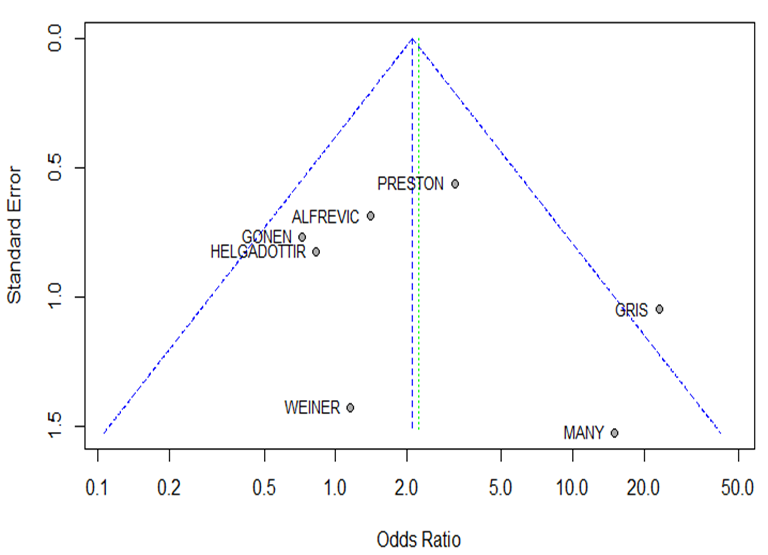

Supplement: Supplementary file 5 — Supplementary file5 (PNG 59 KB) [file 404_2025_7989_MOESM5_ESM.png]

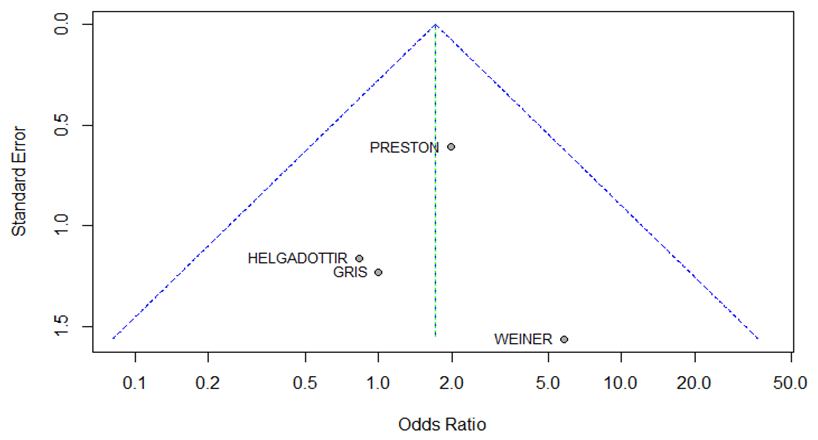

Supplement: Supplementary file 6 — Supplementary file6 (PNG 43 KB) [file 404_2025_7989_MOESM6_ESM.png]

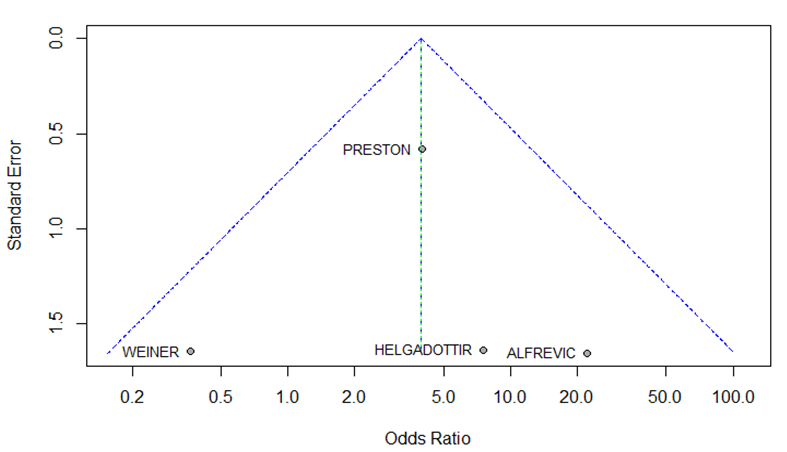

Supplement: Supplementary file 7 — Supplementary file7 (PNG 43 KB) [file 404_2025_7989_MOESM7_ESM.png]

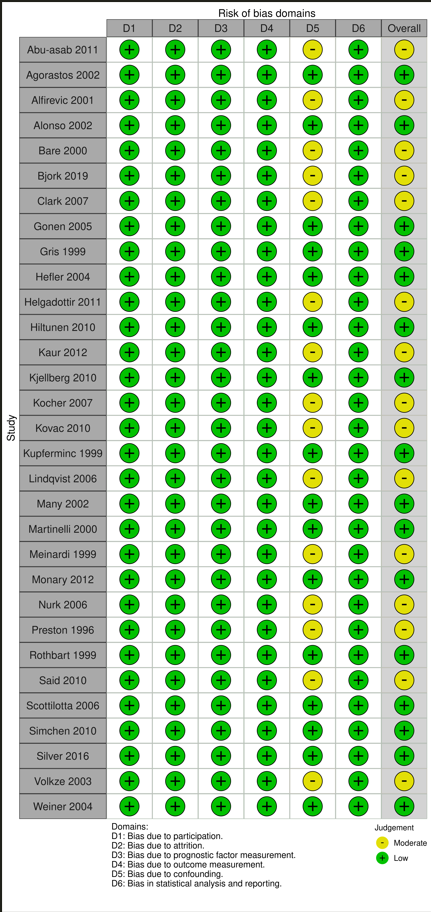

Supplement: Supplementary file 8 — Supplementary file8 (PNG 259 KB) [file 404_2025_7989_MOESM8_ESM.png]

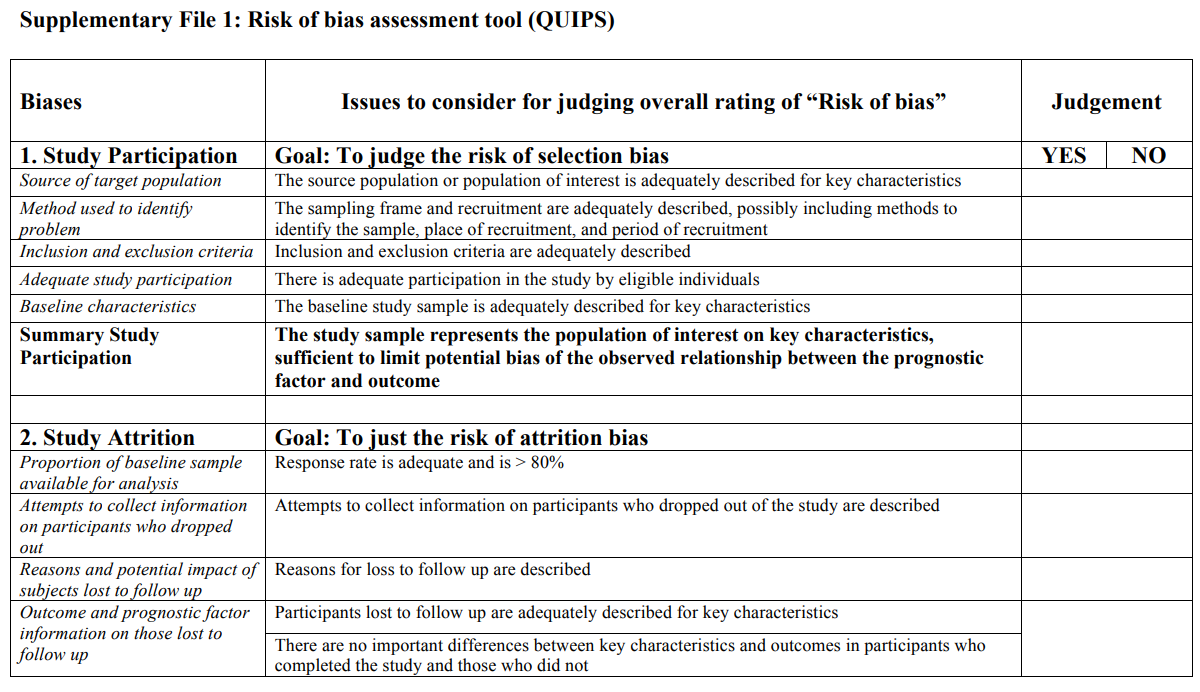

Supplement: Supplementary file 9 — Supplementary file9 (PNG 243 KB) [file 404_2025_7989_MOESM9_ESM.png]

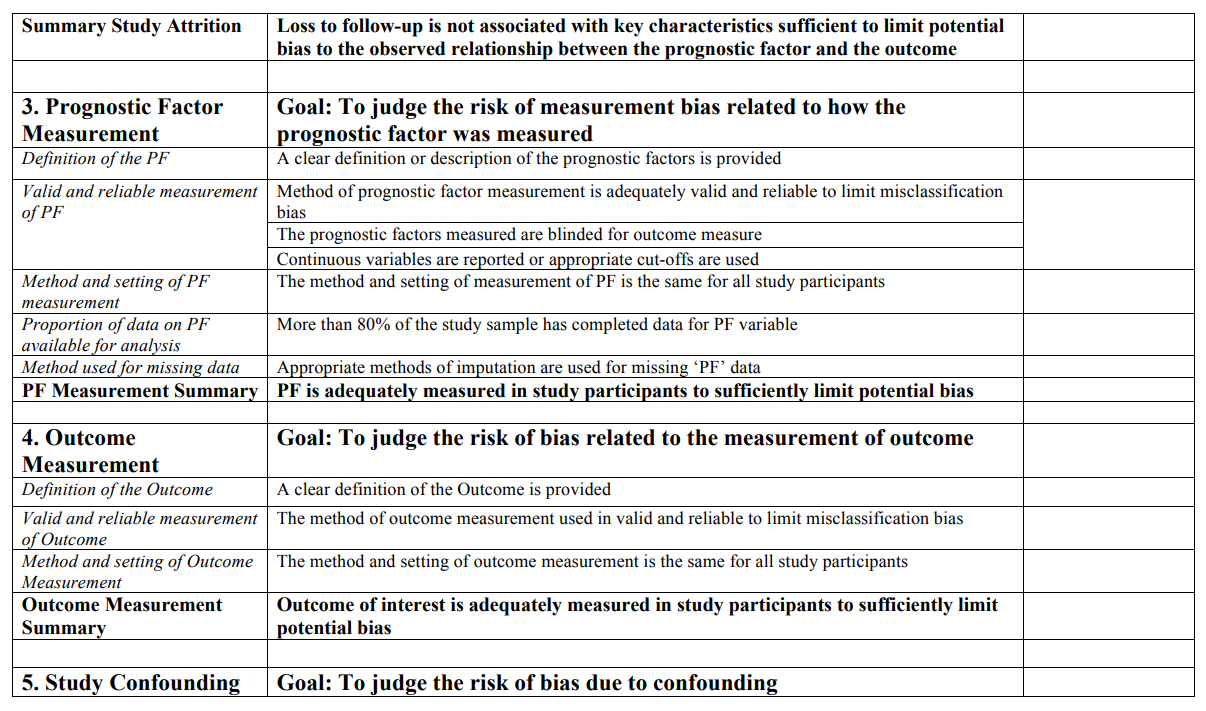

Supplement: Supplementary file 10 — Supplementary file10 (PNG 250 KB) [file 404_2025_7989_MOESM10_ESM.png]

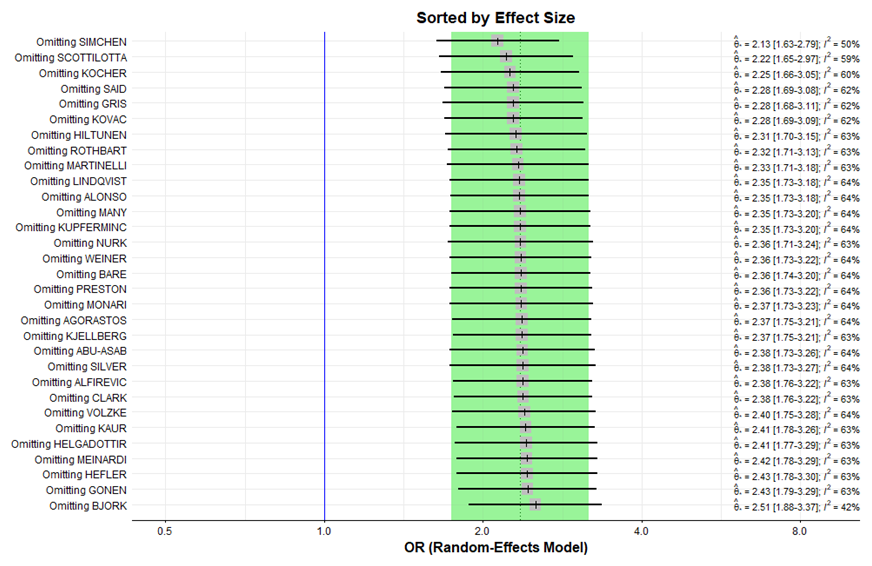

Supplement: Supplementary file 11 — Supplementary file11 (PNG 222 KB) [file 404_2025_7989_MOESM11_ESM.png]

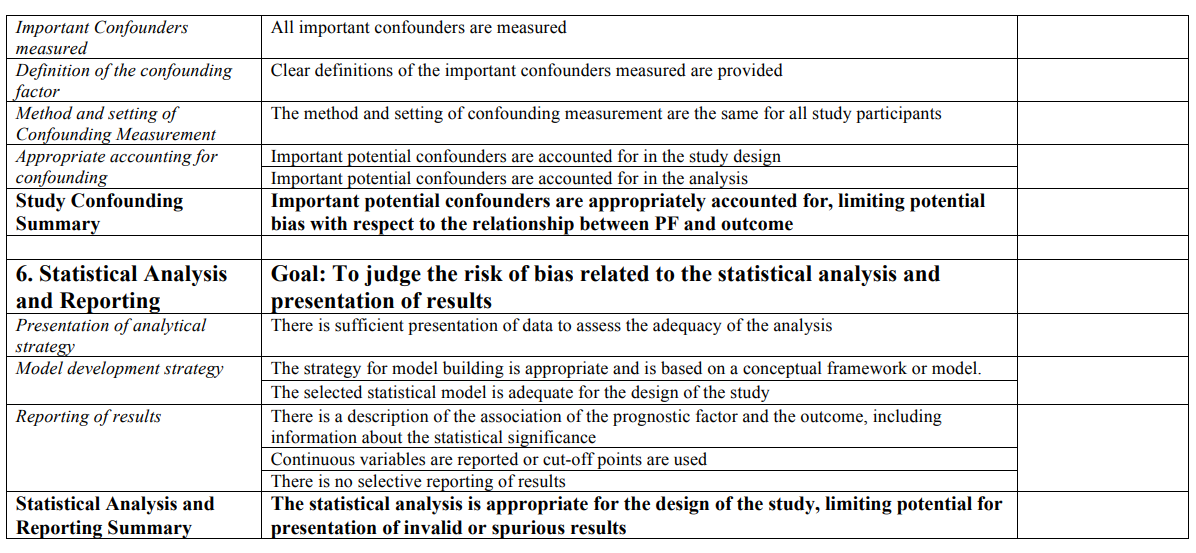

Supplement: Supplementary file 12 — Supplementary file12 (PNG 209 KB) [file 404_2025_7989_MOESM12_ESM.png]

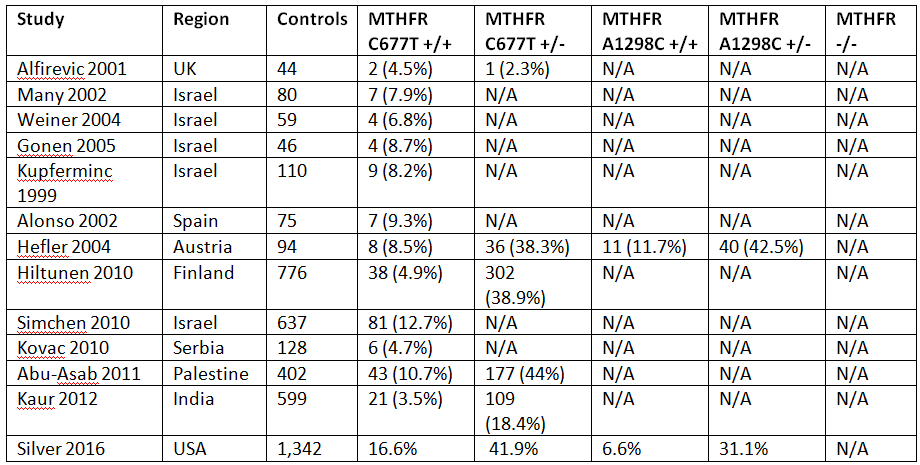

Supplement: Supplementary file 13 — Supplementary file13 (PNG 45 KB) [file 404_2025_7989_MOESM13_ESM.png]

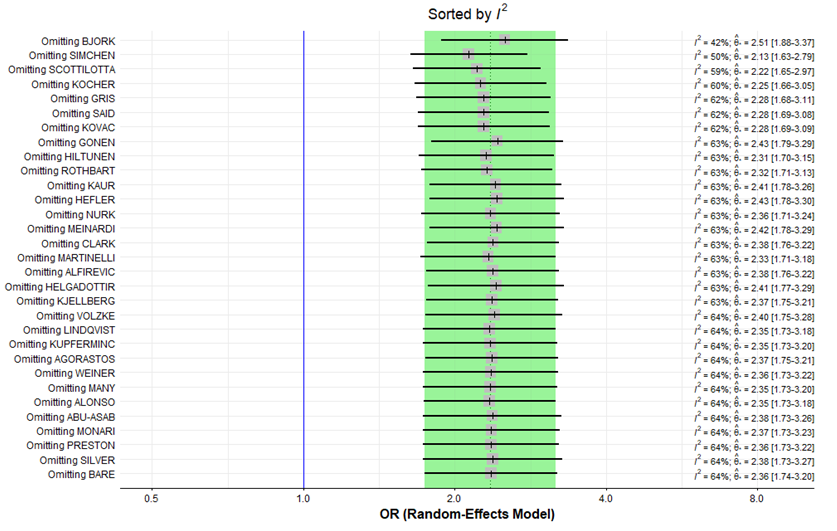

Supplement: Supplementary file 14 — Supplementary file14 (PNG 208 KB) [file 404_2025_7989_MOESM14_ESM.png]

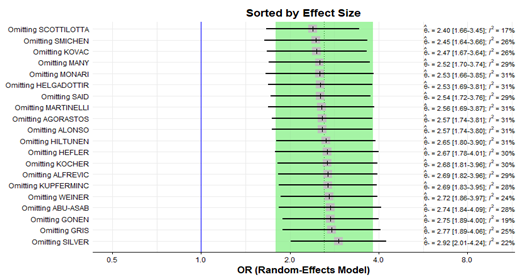

Supplement: Supplementary file 15 — Supplementary file15 (PNG 91 KB) [file 404_2025_7989_MOESM15_ESM.png]

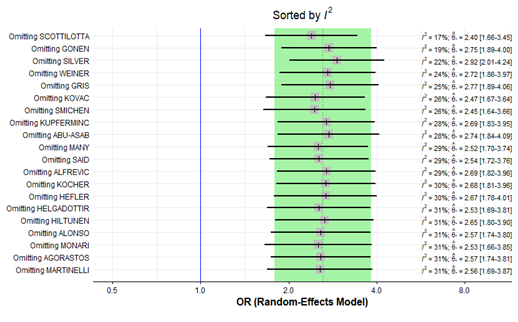

Supplement: Supplementary file 16 — Supplementary file16 (PNG 98 KB) [file 404_2025_7989_MOESM16_ESM.png]

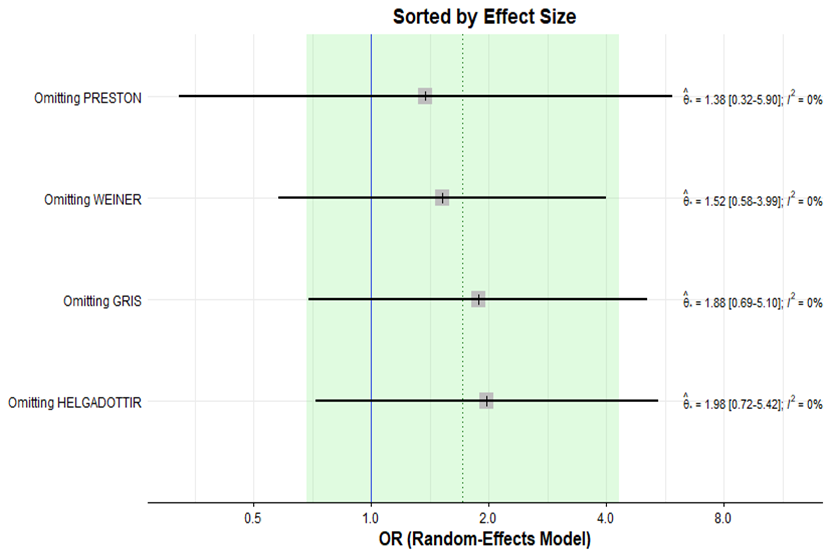

Supplement: Supplementary file 17 — Supplementary file17 (PNG 67 KB) [file 404_2025_7989_MOESM17_ESM.png]

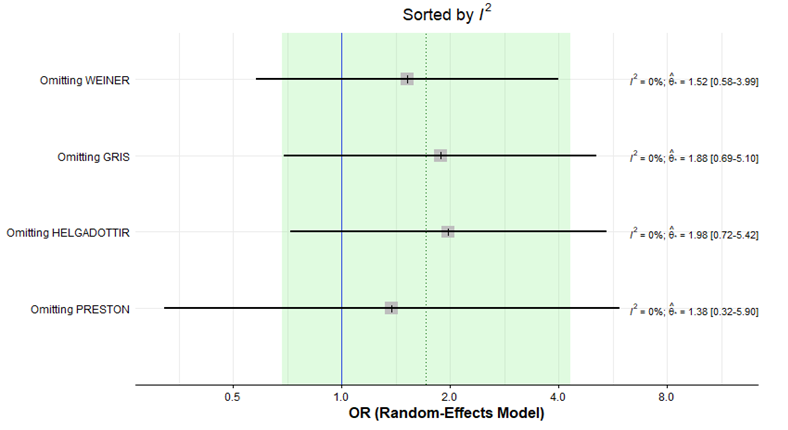

Supplement: Supplementary file 18 — Supplementary file18 (PNG 49 KB) [file 404_2025_7989_MOESM18_ESM.png]

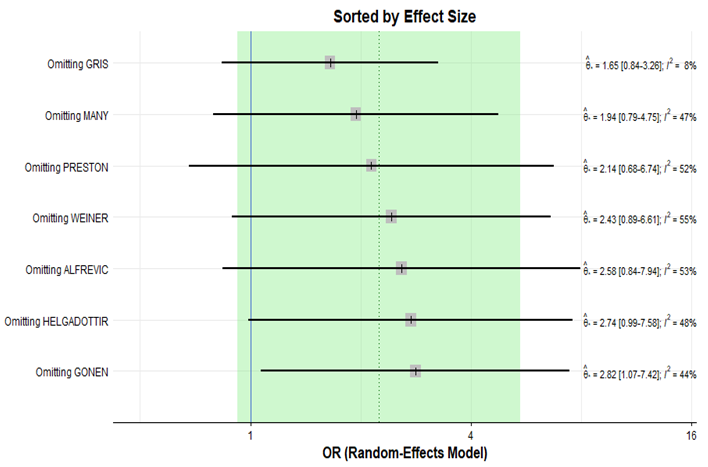

Supplement: Supplementary file 19 — Supplementary file19 (PNG 67 KB) [file 404_2025_7989_MOESM19_ESM.png]

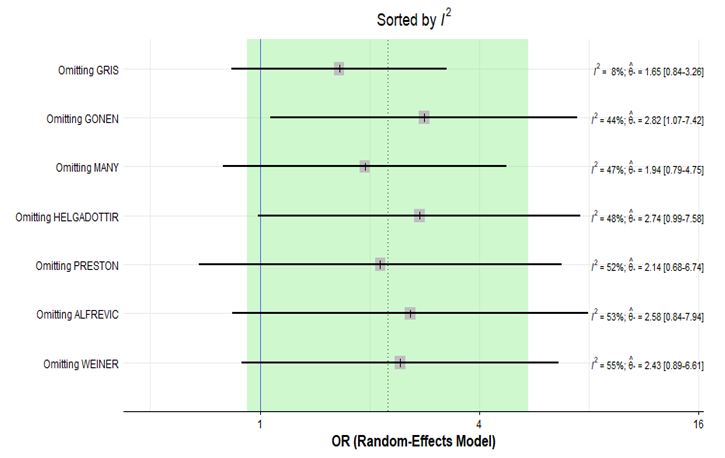

Supplement: Supplementary file 20 — Supplementary file20 (PNG 62 KB) [file 404_2025_7989_MOESM20_ESM.png]

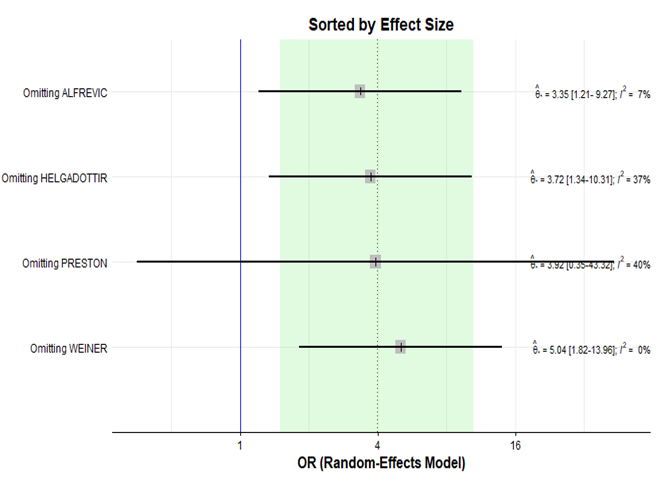

Supplement: Supplementary file 21 — Supplementary file21 (PNG 47 KB) [file 404_2025_7989_MOESM21_ESM.png]
